# Why They Eat What They Eat: Comparing 18 Eating Motives Among Omnivores and Veg^*^ns

**DOI:** 10.3389/fnut.2022.780614

**Published:** 2022-02-21

**Authors:** Markus Müssig, Tamara M. Pfeiler, Boris Egloff

**Affiliations:** ^1^Department of Psychology, Johannes Gutenberg University Mainz, Mainz, Germany; ^2^Leibniz Institute for Resilience Research, Mainz, Germany

**Keywords:** vegan, dietary choices, omnivore, eating motives, vegetarian

## Abstract

While the diets of most people include meat, millions of individuals follow a meat-free diet. But why do people eat what they eat? Here we explored differences and commonalities in the eating motives of omnivores and veg^*^ns (i.e., both vegetarians and vegans). Specifically, we compared mean levels and rank order of 18 eating motives in two samples (Study 1: 294 omnivores, 321 veg^*^ns; Study 2: 112 omnivores, 622 veg^*^ns). We found that omnivores were more motivated than veg^*^ns by the eating motives of *Traditional Eating* and *Habits*, while veg^*^ns were more motivated by *Animal Protection* and *Environmental Protection*. Differences among groups in *Health* were inconsistent across studies. Despite these differences in mean levels, the rank order of the eating motives was very similar: Two of the top four eating motives of both diet groups in both studies were *Liking* and *Health*, while *Social Norms, Social Image*, and *Religion* were among the four least important motives of both groups. Overall, while we did find differences in the absolute importance of certain motives, we also found striking similarities in the relative importance of eating motives, suggesting that including a wide range of eating motives could be beneficial when examining dietary behaviors.

## Introduction

While people following an omnivore diet make up the majority of consumers in Western countries, following a vegetarian or vegan diet is far from a fringe occurrence. In Germany, as of 2020, around 6% of the adult population followed a vegetarian diet, and around 2% followed a vegan diet (1). In the US in 2018, the proportion of adults following a vegetarian diet was around 5%, with around 3% following a vegan diet (2). While these proportions are small compared to omnivores, they represent several million people.

Given the clear delineation regarding food choices between individuals who eat meat (omnivores) and individuals who do not eat meat (termed veg^*^ns by Bagci and Olgun, (3), combining the groups of vegetarians and vegans), the question arises: Why do people eat what they eat? Insights into commonalities and differences in the eating motives of omnivores and veg^*^ns might be important for researchers who study changes in dietary behavior, it might inform studies into motives or barriers and experiences people make when adopting a different diet. This insight may in turn shed light on why people do or do not maintain a new diet. Finally for marketeers, more knowledge about eating motives of omnivores or veg^*^ns might help them to create more targeted interventions or advertisements.

Despite a growing body of research that investigated differences between omnivores and veg^*^ns in socio-demographics (4, 5), personality (6, 7), attitudes (8, 9) and wellbeing (7, 10), much remains to be explored about differences in eating motives among different diet groups. This is in part because research into eating motives has frequently only surveyed vegetarians or vegans (11–14), focused on attitudes toward one specific diet (11, 15–19), or examined eating motives only on a superordinate level such as “health” or “ethics” (20, 21). For these reasons, we analyzed differences and commonalities in 18 eating motives between individuals who eat meat (omnivores) and individuals who do not eat meat (veg^*^ns).

Since the most obvious difference between omnivores and veg^*^ns is the presence of animal products in their diet, ethical concerns regarding animals might be an important eating motive for veg^*^ns. Indeed, dating back to Jabs et al. (13), which identified animal rights as the core ethical concern of veg^*^ns, several additional studies reported animal rights to be a main motivation for veg^*^ns (11, 17, 22, 23). The second core eating motive for veg^*^ns identified by Jabs et al. (13), perceived health benefits as a consequence of a meat-free diet, has also been supported by multiple studies (15, 17, 19, 20, 23–25).

However, an ongoing debate about the conceptualization of ethical eating motives (26) and further research into eating motives has broadened the range of possible relevant eating motives of veg^*^ns. Several studies found environmental concerns or the global impact of meat-eating to be important eating motives for veg^*^ns (17, 18, 20, 22, 27). Other studies found religious beliefs (22), weight control, price (18), or disgust toward meat (12, 25) to be additional eating motives of veg^*^ns.

While research focusing on veg^*^ns is important in understanding dietary choices, we believe it is also important to understand what motivates omnivores to eat what they eat. Previous research identified and examined the 4Ns–it is Normal, Natural, Necessary, and Nice–as four primary justifications for eating meat (16, 28). Renner et al. (29), in a study describing the development of an eating motivation survey, described liking, habits, need and hunger, and health as the most important eating motives for omnivores, while social norms and social image were least important. Graça et al. (30) used eight general consumptions orientations (such as Hedonism or Ethics), as well as seven of the 15 eating motives assessed by Renner et al. (29) to predict the number of meals that were either plant-based, included fish, or included meat in a sample of 1600 Portuguese. Overall, less meat meals as well as more plant-based meals were associated with a higher Ethics orientation, higher Naturalness motivation, higher Health motivation as well as higher Prosumerism orientations. While the study by Graça et al. (30) provides an important basis for a more detailed insight into eating motives, it used only a subset of the eating motives measured by Renner et al. (29) and does not differentiate between different dietary groups.

Only very few studies directly compared eating motives of omnivores and veg^*^ns. In a study of 707 face-to-face interviews (including 43 vegetarians), de Boer et al. (31) found that vegetarians wanted to eat more natural foods^1^ than omnivores did. In a sample including 49 vegetarians and 52 omnivores, using several food-related questionnaires, Dorard and Mathieu (24) found that veg^*^ns were more motivated by health and the natural content of their food, while omnivores were more motivated by weight control. Povey et al. (32) asked 25 meat eaters, 26 meat avoiders (i.e., those excluding meat and poultry but include fish), 34 vegetarians, and 26 vegans to name up to eight beliefs that they assumed were motivations for following different diets. They found that, when asked about their beliefs regarding their own dietary choices, omnivores most frequently named taste, nutrition, and variety, while veg^*^ns mainly named health and ethical reasons. Malek and Umberger (27) asked a sample of 2633 omnivores, 120 vegetarians, and 44 vegans to rate the relative importance of 15 “food choice factors” (p. 2). They found omnivores to be more motivated by price, taste, convenience, and familiarity, while veg^*^ns were more motivated by animal rights and environmental impact; they found no differences in health motivation between these two groups.

These studies suggest that differences in eating motives are not only found regarding health and ethical concerns, but also in other aspects such as taste, naturalness, variety, or price. However, since most studies either asked open questions about eating motives, and thus might have overlooked less salient motives, or used different questionnaires that assessed different–and often just a few–motives, results are rather scattered and far from conclusive. Additionally, most studies described above comprised small samples of veg^*^ns (i.e., samples are mostly only in the double digits). We therefore believe that using a questionnaire that assesses (a) a wide range of eating motives (b) in a comparatively large sample of omnivores and veg^*^ns might be helpful in establishing a foundation for future research, and might aid in bringing together prior research on differences and commonalities in eating motives between omnivores and veg^*^ns. Furthermore, to examine the robustness of findings, we conducted a second study that used a different sample.

In the present studies, we analyzed how similar or different the eating motives of omnivores and veg^*^ns are. To answer these questions, we assessed 18 different eating motives using an extended version of the “The Eating Motives Survey” (29) (*Liking, Habits, Need & Hunger, Health, Convenience, Pleasure, Traditional Eating, Natural Concerns, Sociability, Price, Visual Appeal, Weight Control, Affect Regulation, Social Norms, Social Image, Religion, Animal Protection, Environmental Protection*) in two samples and compared them via rank-order correlations and analyses of mean differences. Study 1 used data gathered from university students and food-related social-media groups, and Study 2 used data gathered via the newsletter of a large vegetarian Non-profit organization. In light of existing literature, we expected veg^*^ns to be more motivated by animal protection, health and environmental protection than omnivores are.

The data reported in this article were part of a larger project about omnivore, vegetarian, and vegan diets that, altogether, comprised three studies. Eating motives were assessed in Studies 1 and 3, and all eating motives are reported here. Since the studies were not preregistered, the findings should be considered exploratory. For a complete list of all measured variables, please see the project page on Open Science Framework (https://osf.io/bj4gv/?view_only=ea5c58e3b1c1488e924c03a9dc2f9eff).

## Study 1

### Method

#### Sample

We recruited participants via a university mailing list and several food-related social media groups in Germany. Participants consented to data collection after being informed about the aims and contents of the survey and were able to quit the survey at any time without penalty. After completion of the online questionnaire, participants could choose between entering a raffle to win a 25-Euro gift certificate, or to donate the same amount to a Non-profit organization. Undergraduate students also received course credit for participating. We did not specify a sample size prior to data collection. Overall, 619 participants (81.4% women) completed the questionnaire. Four participants were excluded from analyses since they did not answer the question about their dietary category. On average, participants were 31.15 years old (*SD* = 11.83).

#### Measures

##### Eating Behavior

To assess eating behavior, participants were asked which dietary category they would place themselves into, with choices being omnivore (*N* = 103), flexitarian (*N* = 167), pescatarian (*N* = 24), ovo-lacto-vegetarian (*N* = 85), ovo-vegetarian (*N* = 5), lacto-vegetarian (*N* = 17), vegan (*N* = 68), and ethically motivated vegan (*N* = 146). All categories were presented with a short description^2^. For our analyses we combined omnivores, flexitarians, and pescatarians into the omnivores group, since all consume some kind of animal meat (*N* = 294, 79.9% women, M_age_ = 29.84, *SD*_age_ = 11.12) and combined ovo-lacto-vegetarians, ovo-vegetarians, lacto-vegetarians, vegans and ethically motivated vegans into the veg^*^ns group (*N* = 321, 84.4% women, M_age_ = 32.37, *SD*_age_ = 12.33).

##### Motives

Motives for participants' current eating behavior were measured via an extended version of the German version of the *The Eating Motivation Survey* [TEMS; (29)]. The TEMS is a 45 item scale measuring 15 eating motives: *Liking* (α = 0.74)*, Habits* (α = 0.71)*, Need & Hunger* (α = 0.60)*, Health* (α = 0.82)*, Convenience* (α = 0.86)*, Pleasure* (α = 0.72)*, Traditional Eating* (α = 0.72)*, Natural Concerns* (α = 0.83)*, Sociability* (α = 0.82)*, Price* (α = 0.80)*, Visual Appeal* (α = 0.66)*, Weight Control* (α = 0.80)*, Affect Regulation* (α = 0.87)*, Social Norms* (α = 0.72), and *Social Image* (α = 0.70). This instrument contains 3 items per motive, all of which use a 7-point Likert-type scale (1 = *never*, 7 = *always*). Confirmatory factor analyses have shown that the 15-factor structure of the TEMS is robust across different samples and countries (33, 34).

To ensure that the original 15-factor model also fits with the characteristics of our data, we conducted the same confirmatory factor analysis with our combined sample and found a largely acceptable model fit (χ^2^ = 1,475.30, *df* = 840, *p* < 0.001; χ^2^/*df* = 1.76, *CFI* = 0.939; *RMSEA* = 0.035). We also conducted multi-group confirmatory factor analysis to test whether mean comparisons on the eating motives between our dietary groups are justified. Between omnivores and veg^*^ns, our data largely showed configural equivalence (χ^2^ = 2,408.50, *df* = 1,680, *p* < 0.001; χ^2^/*df* = 1.43, *CFI* = 0.929; *RMSEA* = 0.027). Restricting the factor loadings to be equal between both groups revealed metric invariance (χ^2^ = 2,437.2, *df* = 1,710, *p* < 0.001; χ^2^/*df* = 1.43, *CFI* = 0.929; *RMSEA* = 0.026; Δχ^2^ = 28.7, Δ*df* = 30, *p* = 0.053; Δ*CFI* = 0; Δ*RMSEA* = −0.001).

Because TEMS did not include other eating motives that might be important to veg^*^ns, we also included self-made items to measure *Religious Motivations* with three items (e.g., I eat what I eat “… because I follow religious rules by doing so”), *Animal Protection* with five items (e.g., “… because animal welfare is important to me in the production of my food”), *Environmental Protection* with six items (e.g., “… because it was produced in an environmentally friendly way”) and *Global Impact* with six items (e.g., “… because it is important to me not to waste resources”). A parallel analysis revealed that a three-factor solution is most suitable for our added items, since most of the items used to measure *Environmental Protection* or *Global Impact* loaded on the same factor (Eigenvalues of observed data: 7.58, 2.17, 1.27, 1.10, 0.63 …; Eigenvalues of simulated data: 1.30, 1.23, 1.19, 1.16, 1.12 …). We reduced the number of items to three per factor, in line with the original TEMS, leaving us with three motives *Religion* (e.g., “… because I follow religious rules by doing so.”; α = 0.80), *Animal Protection* (e.g., “… because animal welfare is important to me in the production of my food.”; α = 0.80) and *Environmental Protection* (e.g., “… because it was produced in an environmentally friendly way.”; α = 0.81). For a list of the final added items, see Supplementary Table 1. The complete list of all added items, as well as the details of our factor analysis, are available from Open Science Framework (https://osf.io/zyu5d/?view_only=6001edaa37a34711886e80585b1f114e).

To test whether these motives are a meaningful addition to the 15 motives of the TEMS, we expanded the original factor model by these motives. An analysis of the resulting 18-factor model with the combined sample revealed a largely acceptable model fit (χ^2^ = 2,097.0, *df* = 1,224, *p* < 0.001; χ^2^/*df* = 1.71, *CFI* = 0.935; *RMSEA* = 0.034). Multi-group confirmatory factor analysis largely showed configural invariance between omnivores and veg^*^ns (χ^2^ = 3,491.7, *df* = 2,448, *p* < 0.001; χ^2^/*df* = 1.43, *CFI* = 0.920; *RMSEA* = 0.026) as well as metric invariance (χ^2^ = 3,551.2, *df* = 2,484, *p* < 0.001; χ^2^/*df* = 1.43, *CFI* = 0.918; *RMSEA* = 0.026; Δχ^2^ = 59.5, Δ*df* = 36, *p* = 0.002; Δ*CFI* = 0.002; Δ*RMSEA* = 0).

#### Analyses

For pairwise comparisons of the 18 eating motives among the diet groups, we conducted *t*-tests. To adjust for multiple testing and the largely exploratory nature of our analyses, we set our significance level to α = 0.001 (35, 36). This specification leads to the fact that only Cohen's *d* effect sizes > 0.3 will become significant for comparisons in both studies. To analyze the similarity of the rank order of eating motives, we performed Spearman correlations of the mean value columns of the respective groups. Data can be retrieved from Open Science Framework at https://osf.io/zyu5d/?view_only=6001edaa37a34711886e80585b1f114e.

### Results

Omnivores were most motivated–indicated by the highest values in the respective columns in Table 1–by *Liking, Needs & Hunger, Health*, and *Habits*, while veg^*^ns were most motivated by *Liking, Health, Animal Protection*, and *Need & Hunger*. Both omnivores and veg^*^ns were least motivated by *Religion, Social Image, Affect Regulation*, and *Social Norms*. Spearman correlations of the mean-value columns showed a rank-order similarity of rho = 0.87.

**Table 1:** Study 1: Mean differences in eating motives between omnivores and veg*ns.

	**Total sample**		**Omnivores**		Veg^*^ns				
**Motive**	***M***	***SD***	***M***	***SD***	***M***	***SD***	***t***	***p***	***d***
Liking	6.18	0.76	6.20	0.75	6.16	0.78	0.66	0.507	0.05
Habits	4.54	1.30	4.86	1.23	4.25	1.29	6.04	<0.001	**0.48**
Need & Hunger	5.55	0.91	5.57	0.86	5.52	0.95	0.69	0.493	0.06
Health	5.49	1.14	5.23	1.17	5.73	1.05	−5.54	<0.001	**–0.45**
Convenience	4.67	1.38	4.71	1.46	4.63	1.30	0.69	0.488	0.06
Pleasure	4.79	1.23	4.83	1.24	4.77	1.23	0.59	0.554	0.05
Traditional Eating	3.28	1.50	4.09	1.34	2.55	1.22	14.95	<0.001	**1.20**
Natural Concerns	5.09	1.43	4.72	1.54	5.43	1.23	−6.31	<0.001	**–0.51**
Sociability	4.09	1.56	4.37	1.59	3.83	1.50	4.33	<0.001	**0.35**
Price	3.68	1.47	3.83	1.51	3.54	1.43	2.41	0.016	0.20
Visual Appeal	2.94	1.25	3.09	1.30	2.80	1.18	2.85	0.005	0.23
Weight Control	3.66	1.54	3.67	1.61	3.66	1.48	0.14	0.890	0.01
Affect Regulation	2.48	1.48	2.41	1.45	2.54	1.51	−1.12	0.261	-0.09
Social Norms	2.21	1.16	2.47	1.24	1.97	1.03	5.39	<0.001	**0.44**
Social Image	1.70	0.88	1.74	0.90	1.66	0.86	1.03	0.303	0.09
Religion	1.15	0.63	1.17	0.66	1.13	0.61	0.69	0.490	0.06
Animal Protection	5.09	1.64	4.43	1.55	5.70	1.47	−10.44	<0.001	**–0.84**
Environmental Protection	4.55	1.53	3.74	1.38	5.29	1.26	−14.56	<0.001	**–1.18**

N (Omnivores) = 294, N (Veg^*^ns) = 321. Effect sizes printed in bold are significant at p < 0.001. Eating motives were answered on a 7-point Likert-type scale (1 = never, 7 = always). In control analyses including age as a covariate, the results were virtually identical.

*T*-tests (Table 1) showed that, compared to veg^*^ns, omnivores were more motivated by *Traditional Eating* (*d* = 1.20), *Habits* (*d* = 0.48), *Social Norms* (*d* = 0.44), and *Sociability* (*d* = 0.35). Veg^*^ns, on the other hand, were more motivated than omnivores by *Environmental Protection* (*d* = −1.18), *Animal Protection* (*d* = −0.84), *Natural Concerns* (*d* = −0.51), and *Health* (*d* = −0.45).

### Discussion

Overall, omnivores and veg^*^ns showed differences in eight eating motives and similarities in ten eating motives. We assumed that veg^*^ns were more motivated by animal protection, health and environmental protection than omnivores, which was in line with our results. However, these were not the only differences, since we also found veg^*^ns to be more motivated by a desire for natural foods, while omnivores were more motivated by traditions, habits or norms. Considering the rank correlation of rho = 0.87, despite differences in specific eating motives, the relative importance of the assessed eating motives was very similar.

## Study 2

In Study 2, we aimed to replicate the results from Study 1 to identify commonalities and differences between diet groups that are consistent across different samples with different characteristics. We also changed our recruiting strategy to survey even more veg^*^ns.

### Method

#### Sample

We recruited participants via the newsletter of one of the largest German Non-profit vegetarian associations, *ProVeg International*. *ProVeg International* did not commission the survey and we did not receive financial compensation. Participants consented to data collection after being informed about the aims and contents of the survey and could withdraw from the survey at any time without penalty. Similar to Study 1, participants could choose to either enter a raffle to win a 50-euro gift certificate or to donate the same amount to a Non-profit organization. Overall, 749 participants (77.6% women) completed the questionnaire. Fifteen participants were excluded from our analyses since they did not answer the question about their dietary category. On average, participants were 44.37 years old (*SD* = 14.49).

#### Measures

##### Eating Behavior

Eating behavior was assessed in the same way as in Study 1. The sample included 7 omnivores, 64 flexitarians, 41 pescatarians, 148 ovo-lacto-vegetarians, 17 ovo-vegetarians, 43 lacto-vegetarians, 136 vegans, and 278 ethically motivated vegans. Similar to Study 1, we combined omnivores, flexitarians and pescatarians into the category of omnivores (*N* = 112, 77.6% women, M_age_ = 45.52, *SD*_age_ = 14.87); ovo-lacto-, ovo-, lacto-vegetarians, vegans and ethically motivated vegans into the category of veg^*^ns (*N* = 622, 78.9% women, M_age_ = 44.16, *SD*_age_ = 14.42).

##### Motives

We assessed the same 18 motives as in Study 1, but–due to time constraints–reduced the number of items to one item per motive. Motives were assessed by the following items: *Liking* “… because it tastes good”, *Habits* “… because I am used to it”, *Need & Hunger* “… to get full”, *Health* “… to provide me with important nutrients”, *Convenience* “… because I can do it with low effort”, *Pleasure* “… because I enjoy it”, *Traditional Eating* “… because it is part of certain situations”, *Natural Concerns* “… because it is natural”, *Sociability* “… because it is sociable”, *Price* “… because it is cheap”, *Visual Appeal* “… because it looks inviting”, *Weight Control* “… because I want to control my weight”, *Affect Regulation* “… to feel good”, *Social Norms* “… because the opinion of others is important to me”, *Social Image* “… because it is trendy”, *Religious Motivations* “… because my religion requires me to”, *Animal Protection* “… because animal rights are important to me”, *Environmental Protection* “… because environmental protection is important to me”. All items were determined by researcher discussion and were scored on a Likert-type scale ranging from 1 to 11.

#### Analyses

We conducted the same analyses as in Study 1 and applied the same significance level (α = 0.001). Data can be retrieved from Open Science Framework at https://osf.io/zyu5d/?view_only=6001edaa37a34711886e80585b1f114e.

### Results

Omnivores were most motivated by *Health, Liking, Animal Protection*, and *Natural Concerns*, while Veg^*^ns were most motivated by *Animal Protection, Environmental Protection, Health*, and *Liking*. Omnivores were least motivated by *Religion, Social Image, Affect Regulation*, and *Social Norms*, while Veg^*^ns were least motivated by *Religion, Social Image, Social Norms*, and *Traditional Eating*. Spearman correlations of the mean-value columns showed a rank-order similarity of rho = 0.96.

*T*-tests (Table 2) showed that, compared to veg^*^ns, omnivores were more motivated by *Traditional Eating* (*d* = 0.65), *Weight Control* (*d* = 0.42), *Habits* (*d* = 0.37), and *Price* (*d* = 0.36). Veg^*^ns were more motivated than omnivores by *Animal Protection* (*d* = −0.65), and *Environmental Protection* (*d* = −0.46).

**Table 2:** Study 2: Mean differences in eating motives between omnivores and veg*ns.

	**Total sample**		**Omnivores**		Veg^*^ns				
**Motive**	***M***	***SD***	***M***	***SD***	***M***	***SD***	***t***	***p***	***d***
Liking	8.38	0.76	8.53	1.88	8.35	2.15	0.63	0.529	0.07
Habits	4.27	1.30	5.18	2.27	4.10	2.28	3.62	<0.001	**0.37**
Need & Hunger	7.33	0.91	7.53	2.38	7.28	2.78	0.67	0.501	0.07
Health	8.78	1.14	8.70	1.83	8.80	1.90	−0.34	0.732	−0.04
Convenience	5.68	1.38	6.60	2.47	5.52	2.43	3.38	0.001	0.35
Pleasure	6.00	1.23	6.30	2.45	5.95	2.60	1.02	0.308	0.11
Traditional Eating	2.55	1.50	3.83	2.37	2.32	1.52	5.05	<0.001	**0.65**
Natural Concerns	8.10	1.43	8.15	2.03	8.08	2.23	0.25	0.802	0.02
Sociability	4.32	1.56	5.07	2.22	4.18	2.33	2.84	0.005	0.30
Price	4.32	1.47	5.17	2.20	4.17	2.05	3.60	<0.001	**0.36**
Visual Appeal	3.53	1.25	4.08	2.12	3.43	1.97	2.38	0.018	0.24
Weight Control	3.87	1.54	4.95	2.65	3.67	2.38	3.81	<0.001	**0.42**
Affect Regulation	3.23	1.48	3.45	2.12	3.20	2.07	0.86	0.391	0.09
Social Norms	2.12	1.16	2.53	1.70	2.03	1.32	2.07	0.040	0.24
Social Image	2.02	0.88	2.23	1.45	1.97	1.18	1.39	0.166	0.14
Religion	1.32	0.63	1.42	0.97	1.30	0.60	0.77	0.440	0.09
Animal Protection	9.67	1.64	8.28	2.42	9.92	1.72	−5.35	<0.001	–**0.65**
Environmental Protection	8.85	1.53	7.88	2.18	9.03	1.77	−4.03	<0.001	–**0.46**

N (Omnivores) = 112, N (Veg^*^ns) = 622. Effect sizes printed in bold are significant at p < 0.001. Eating motives were answered on a Likert-type scale ranging from 1 to 11 (1 = never, 11 = always). In control analyses including age as a covariate, the results were virtually identical.

### Discussion

The pattern of similarities and differences between omnivores and veg^*^ns in Study 2 was almost identical to that of Study 1 (differences in *Animal Protection, Environmental Protection, Traditional Eating, Habits*). However, the difference in the importance of *Health* between omnivores and veg^*^ns found in Study 1 could not be replicated. This observation, combined with the rank correlation of eating motives of rho = 0.96 and the fact that only 7% of the people in the omnivores group were strict omnivores (with the other 93% being pescatarians or flexitarians), suggests that the omnivores in Study 2 were far more similar to the veg^*^ns than the omnivores in Study 1 were. This is most likely explained either by our recruiting strategy via the newsletter of a vegetarian Non-profit organization (i.e., any omnivore readers shared many beliefs held by vegetarians) or by the wording of the single *Health* item in Study 2 (“I eat what I eat to provide me with important nutrients”) that likely measures only a narrower range of *Health* motivation. We also found unexpected group differences for the motives of *Convenience, Price*, and *Weight Control*. However, despite these differences, the pattern of commonalities and differences between diet groups found in Study 2 was largely the same as in Study 1. Thus, despite our shortened questionnaire and what was probably a more homogenous sample overall, the results of Study 2 largely match those of Study 1.

## General Discussion

### Differences and Commonalities Between Omnivores and Veg^*^ns

Since most of the prior studies only provided small sample sizes of veg^*^ns or measured only a few motives that they compared between groups, we wanted to survey a large number of veg^*^ns and measure a wide range of eating motives in two independent studies. Overall, we found that omnivores were more motivated than veg^*^ns by the eating motives of *Traditional Eating* and *Habits*, while veg^*^ns were more motivated by *Animal Protection* and *Environmental Protection*. Differences among groups in *Health* were inconsistent across studies. Despite these differences in mean levels, the rank order of the eating motives was very similar: Two of the top four eating motives of both diet groups in both studies were *Liking* and *Health*, while *Social Norms, Social Image*, and *Religion* were among the four least important motives of both groups.

The long-held assumption that the treatment of animals and other ethical concerns (i.e., environmental protection) are among the main eating motives for veg^*^ns, and, further, that these motivations are more important to veg^*^ns than they are to omnivores, is largely supported by our results. Additionally, *Traditional Eating*, a motive largely overlooked (with the exception of one of the 4N, *Normal* (16, 28), which resembles traditions), was among the top three differences in eating motives between omnivores and veg^*^ns in both studies. Considering that omnivores were also more motivated by *Habits* than veg^*^ns, our findings suggest that the most important factors distinguishing omnivores and veg^*^ns are ethical concerns (i.e., *Animal Protection, Environmental Protection*) and traditional values (i.e., *Habits, Traditional Eating*). Because meat consumption can be understood as a tradition (e.g., many festive meals revolve around it), it is not surprising that *Traditional Eating* is among the top eating motives of omnivores. At the same time, this traditional way of eating is challenged by veg^*^ns on moral arguments such as *Animal Rights* or *Environmental Protection* (14, 29), it seems plausible that veg^*^ns give less weight to traditions and habits, but comparatively more weight to moral or ethical arguments.

However, despite several studies finding *Health* as an important eating motive for veg^*^ns (15, 17, 19, 20, 23–25), our results are somewhat inconclusive. It was only more important to veg^*^ns than to omnivores in Study 1, but not in Study 2. While there are important limitations due to our methods (what we measured as *Health* might differ between Study 1 and Study 2), our inconclusive results are more in line with findings of Trethewey & Jackson (37) who found that while health concerns differed between omnivores and veg^*^ns, health concerns did not predict meat consumption. It seems that personal health is a less important factor in differentiating omnivores from veg^*^ns than moral or traditional motives. Considering that health is a frequently found motive for vegetarians (but also for omnivores), we believe that these inconsistencies deserve attention in future research. It might be interesting to see how perceived health benefits of certain foods such as meat or vegetables change over time and how these changes are linked to an omnivore or veg^*^n diet.

Some other differences, such as in *Price* and *Weight Control*, were also inconsistent across samples, and need to be replicated by additional studies to clarify whether or not they are useful in distinguishing omnivores from veg^*^ns.

Despite such differences, the relative importance of eating motives between veg^*^ns and omnivores was surprisingly similar (0.87 < rho < 0.96). For example, *Religion, Social Image*, and *Social Norms* were among the bottom four eating motives in both studies. Especially since perceived social norms are important predictors of behavior (38), we expected them to play a more important role. However, this observation might be the result of social factors being less available to introspection (19) or, contrary, of participants wanting to downplay the role of social factors for their decision-making. The most important motives were also rather similar: *Liking* and *Health* were among the top four eating motives for omnivores and veg^*^ns alike in both studies. Concerning omnivores, these results are in line with results from Rosenfeld & Tomiyama (19) showing that taste and health concerns trump anticipated stigma, perceived financial cost, or convenience as barriers to vegetarianism. Additionally, results from Piazza et al. (28) show that Nice and Necessary (subscales of the 4N resembling liking and health) were significant predictors for commitment to eating meat while Normal and Natural were not.

This means that researchers studying different dietary choices who ask a sample of veg^*^ns in an interview, “Why do you eat a vegetarian (or vegan) diet?” and receive answers such as “Because I think it is healthy” might overlook other important eating motives such as *Liking* which is probably less salient to veg^*^ns (i.e., not easily accessible to introspection). Omnivores, however, seem to be aware that *Liking* is important to them (19). While we already addressed *Health*, our results regarding *Liking* seem especially important to us –namely that it was one of the most important eating motives for both groups with no difference between them. For omnivores, *Liking* might be a strong incentive to justify meat-eating, especially to people who also have ethical concerns regarding meat-eating. It also implies that individuals who do not enjoy a certain diet might be less inclined to adhere to this diet, despite it being more in line with their moral or social values. Similarly, a person who really likes a plant-based diet might find it easier to follow this diet for a long time despite traditional values or perceived diverging social norms.

### Limitations and Future Directions

While we achieved our goal of reaching a large number of veg^*^ns in an age-diverse sample, our samples were overwhelmingly female (Study 1: 81.4%; Study 2: 77.6%). Additionally, our recruiting strategies, via food-related social-media groups and the newsletter of a vegetarian Non-profit organization, likely mean that our sample overall is not representative of the German population. Since 65% of the people in Study 1 and 93% of the people in Study 2 were not strict omnivores, we assume that the omnivores group in our samples–especially in Study 2–was more similar to veg^*^ns than in population-representative samples and, consequently, that differences in the general population might be larger than those found in our studies.

Second, our extension of the TEMS by adding items and categories, despite the factor analyses we performed, did not follow classic techniques of questionnaire development and validation, making this assessment more exploratory in nature. Together, the changes we made between Study 1 and Study 2 likely attenuated all differences we found, and might also had an effect on the rank order of eating motives.

Third, an issue that we could not resolve was whether or not we actually assessed eating motives, or not some other factor. What does or does not qualify as an eating motive is subject to debate (26), and some of our assessed eating motives might also qualify as barriers, depending on one's perspective and definition. For example, an omnivore might be motivated to adhere to an omnivorous diet because of *Habits*, but this same factor also qualifies as a barrier to adopting a veg^*^n diet. Since we were only interested in eating motives of current dietary behavior, we stuck to the wording used by Renner et al. (29). Additionally, some of the eating motives might serve as rationalizations for behavior and hence might not be “true” motives, which we were not able to differentiate.

Lastly, as is the case with all questionnaire data, self-reporting bias might limit our conclusions. Participants might have been hesitant to admit that their diet is motivated by *Social Norms, Social Image* or *Weight Loss*. Future studies therefore might find it interesting to include implicit measures of eating motives that are constructed to circumvent self-reporting bias. As an implicit measure of how healthy respondents assess meat compared to other foods, researchers might show pictures of different foods and request respondents to assess their “healthiness”. Alternatively, researchers could ask for personal motives and compare them to reported eating motives to detect areas that might be prone to biases.

Despite these limitations, we believe that research into changes in dietary behavior should consider these results, as omnivores might refrain from adopting a veg^*^n diet simply because they do not like (or believe they do not like) plant-based foods or are not convinced that a veg^*^n diet might be healthy. Furthermore, since both omnivores and veg^*^ns ranked *Health* among their most important eating motives, consumers likely do not change their diet because their health motivation changes. It seems more likely that consumers choose to adopt the diet they *perceive* to be the most beneficial for their health. Longitudinal studies are necessary to better understand these processes.

## Conclusion

We believe our data are meaningful to researchers in the field of eating motives, since we provide comparisons of a wide range of 18 different eating motives between omnivores and veg^*^ns. Focusing on the differences in eating motives between dietary groups might overemphasize differences and overlook similarities, since we found high rank-order correlations of eating motives between omnivores and veg^*^ns (0.87 < rho < 0.96). Both diet groups in our two studies report that they eat what they eat because they like it and because they think it is healthy for them; very few seem to care about their *Social Image* or *Social Norms*. Moreover, the differences in eating motives between different dietary groups that we found in both samples (*Habits, Traditional Eating, Animal Protection, Environmental Protection*), and differences we consistently did not find (e.g., *Liking, Need & Hunger, Visual Appeal*, or *Social Image*) might help explain why people do or do not change their dietary behavior. A further clarification of the inconsistencies we found between both samples (e.g., *Health, Price*, or *Weight Control*) would be desirable.

Additionally, our results have methodical implications, since research that exclusively asks veg^*^ns about their motivations for following their current diet and that finds, for example, that health is the most important eating motive for veg^*^ns likely falls short in two aspects: (a) health might also be the most important eating motive of omnivores and is therefore not a unique feature of veg^*^ns, and (b) there might be other, probably more mundane, motives such as *Liking* that are less salient. Therefore, researchers might find it helpful to also assess other diet groups and use questionnaires that include a wider range of eating motives for more conclusive results. Ideally, this research would be done in large, representative samples for more generalizable results. Considering these results in further research might increase our understanding of dietary behaviors and the processes that lead to changes in these behaviors.

## Data Availability Statement

The datasets presented in this study are freely available in online repositories and can be found below: https://osf.io/zyu5d/?view_only=6001edaa37a34711886e80585b1f114e.

## Ethics Statement

Ethical review and approval was not required for the study on human participants in accordance with the local legislation and institutional requirements. The patients/participants provided their written informed consent to participate in this study.

## Author Contributions

MM: conceptualization, data curation, formal analysis, writing—original draft, and writing—review and editing. TP: data curation, investigation, and writing—review and editing. BE: conceptualization, project administration, supervision, writing—original draft, and writing—review and editing. All authors contributed to the article and approved the submitted version.

## Conflict of Interest

The authors declare that the research was conducted in the absence of any commercial or financial relationships that could be construed as a potential conflict of interest.

## Publisher's Note

All claims expressed in this article are solely those of the authors and do not necessarily represent those of their affiliated organizations, or those of the publisher, the editors and the reviewers. Any product that may be evaluated in this article, or claim that may be made by its manufacturer, is not guaranteed or endorsed by the publisher.

## Footnotes

^1^de Boer et al. (31) measured this with the item “I prefer to prepare food myself because I want to eat everything as pure as possible”.

^2^Omnivore = regularly eats different kinds of animal products; Flexitarian = usually follows a vegetarian diet but occasionally eats meat; Pescatarian = excludes red meat and poultry but eats fish; Ovo-lacto = includes egg and milk products; Ovo = excludes milk products but includes egg products; Lacto = excludes egg but includes milk products; Vegan = excludes animal products from the diet but buys leather or wool products; Ethically motivated vegan = does not buy any animal products, including leather, wool, …
