# Supplementary material for: Why They Eat What They Eat: Comparing 18 Eating Motives Among Omnivores and Veg*ns
Source: Front Nutr. 2022 Feb 21;9:780614. doi: 10.3389/fnut.2022.780614 (PMC8899081; doi:10.3389/fnut.2022.780614)
Supplement: Supplementary file 1 [file Table_1.pdf]

**Supplementary Table 1**<br>Original (German) and translated items of the three added eating motives.	
English	German
I eat what I eat because…	Ich esse das, was ich esse, …
**Religion**<br>… because I follow religious rules by doing so<br>… because my religion requires me to abstain from certain foods<br>… because my religion dictates the consumption of certain foods	**Religion**<br>...weil ich damit religiöse Vorschriften befolge.<br>...weil meine Religion von mir den Verzicht auf bestimmte Lebensmittel verlangt.<br>...weil durch meine Religion der Verzehr bestimmter Lebensmittel vorgegeben ist.
**Animal Protection**<br>… because animal welfare is important to me in the production of my food<br>… because important animal welfare standards are adhered to by doing so<br>… because as much attention as possible is paid to the needs of animals by doing so	**Tierschutz**<br>...weil mir artgerechte Tierhaltung bei der Produktion meiner Lebensmittel wichtig ist.<br>...weil dafür wichtige Tierschutzstandards eingehalten werden.<br>...weil dafür möglichst stark auf die Bedürfnisse von Tieren geachtet wird.
**Environmental Protection**<br>… because it was produced in an environmentally friendly way<br>… because little drinking water is used to produce it<br>… because it is important to me not to waste resources	**Umweltschutz**<br>...weil es auf umweltfreundliche Art produziert wurde.<br>**…** weil dafür wenig Trinkwasser verbraucht wird.<br>...weil es mir wichtig ist, keine Ressourcen zu verschwenden.
Note. Detailed information about the entire questionnaire as well as the factor analysis are available from Open Science Framework (https://osf.io/zyu5d/?view_only=6001edaa37a34711886e80585b1f114e).	
